# Cytogenetics of *Melitoma segmentaria* (Fabricius, 1804) (Hymenoptera, Apidae) reveals differences in the characteristics of heterochromatin in bees

**DOI:** 10.3897/CompCytogen.v8i3.7510

**Published:** 2014-08-14

**Authors:** Maykon Passos Cristiano, Talitta Guimarães Simões, Denilce Meneses Lopes, Silvia das Graças Pompolo

**Affiliations:** 1 Departamento de Biodiversidade, Evolução e Meio Ambiente, Universidade Federal de Ouro Preto, Morro do Cruzeiro, Ouro Preto, Minas Gerais, Brazil, 35400-000; 2 Departamento de Biologia Geral, Universidade Federal de Viçosa, Avenida Peter Henry Rolfs s/n, Viçosa, Minas Gerais, Brazil, 36570-000

**Keywords:** cytogenetic characterization, heterochromatin, fluorochromes, solitary bees, karyotype evolution

## Abstract

To date, more than 65 species of Brazilian bees (of the superfamily Apoidea) have been cytogenetically studied, but only a few solitary species have been analyzed. One example is the genus *Melitoma* Lepeletier & Serville, 1828, for which there is no report in the literature with regard to cytogenetic studies. The objective of the present study is to analyze the chromosome number and morphology of the species *Melitoma segmentaria* (Fabricius, 1804), as well as to determine the pattern of heterochromatin distribution and identify the adenine–thymine (AT)- and guanine–cytosine (GC)-rich regions. *Melitoma segmentaria* presents chromosome numbers of 2n=30 (females) and n=15 (males). With C-banding, it is possible to classify the chromosomes into seven pseudo-acrocentric pairs (A^M^), seven pseudo-acrocentric pairs with interstitial heterochromatin (A^Mi^), and one totally heterochromatic metacentric pair (M^h^). Fluorochrome staining has revealed that heterochromatin present in the chromosomal arms is rich in GC base pairs (CMA_3_^+^) and the centromeric region is rich in AT base pairs (DAPI^+^). The composition found for *Melitoma* diverges from the pattern observed in other bees, in which the heterochromatin is usually rich in AT. In bees, few heterochromatic regions are rich in GC and these are usually associated with or localized close to the nucleolus organizer regions (NORs). Silver nitrate impregnation marks the heterochromatin present in the chromosome arms, which makes identification of the NOR in the chromosomes impossible. As this technique reveals proteins in the NOR, the observation that is made in the present study suggests that the proteins found in the heterochromatin are qualitatively similar to those in the NOR.

## Introduction

The genus *Melitoma* Lepeletier & Serville, 1828 belongs to the tribe Emphorini and has 10 described species. These are solitary bees, which nest in cavities in the soil, are typically gregarious, and are distributed from Mexico to Argentina (Mamede-Filho et al. 1991). These bees are closely associated with a particular plant, *Ipomoea* sp. (Convolvulaceae), and the presence or absence of this plant generally defines their distribution (Schlising 1970).

Cytogenetic studies of Brazilian bees are more common in the eusocial species belonging to the tribe Meliponini. These studies were initiated by Kerr (1948). Since then, more than 28 genera and 65 species have been analyzed. The haploid chromosome number in the bees of this tribe ranges from eight to eighteen, where n=17 is the predominant number (Rocha et al. 2003).

Little cytogenetic information has been obtained for solitary bees. In the literature, only some cytogenetic information for the species of the genus *Eufriesea* Cockerell, 1908 (Gomes et al. 1998), *Euglossa* Latreille, 1802 (Maffei et al. 2001, Fernandes et al. 2013), *Ceratina* Latreille, 1802, *Xylocopa* Latreille, 1802, and *Pithitis* Klug, 1807 (Hoshiba and Imai 1993) is found. The same is true for the genus *Melitoma*, where none of the ten species are cytogenetically characterized.

Cytogenetic studies are important because they contribute a great deal to the understanding of evolutionary mechanisms that contribute to the changes in genome organization. By using different chromosomal banding techniques we can study different chromosomal characters that can be used to solve taxonomic issues. A simple karyotype analysis allows the observation of variations, such as, differences in chromosome number, size, and specific base pair composition of the DNA, enhancing our knowledge of the evolution and phylogenetic relationships of different species (White 1973, Imai et al. 1994, Sumner 2003).

The “minimum interaction hypothesis” proposed by Imai et al. (1988), is the most commonly used mechanism to explain chromosome diversity and evolution in Hymenoptera, mainly in ants and bees (Rocha et al. 2003). According to this hypothesis, karyotype evolution is biased toward an increase in acrocentric chromosomes, thereby reducing the risk of deleterious rearrangements, due to a decrease of the potential contact among the chromosomes in the nucleus. Although occasional fusions that decrease the chromosome number are not excluded by “the minimum interaction hypothesis”, fissions appear more likely. However, Fernandes et al. (2013) and Cardoso et al. (2014) based on the studies of solitary bees and ants, have suggested that other mechanisms may be involved in the karyotype evolution of social Hymenoptera.

In this context, the aim of the present study is to analyze the karyotype, including the chromosome number and morphology, distribution pattern of the heterochromatin, and richness of composition of the AT and GC base pairs, of the solitary bee species *Melitoma segmentaria*, thereby contributing to an increase in the cytogenetic knowledge of this genus and providing interesting new insights about the genome organization in these bees.

## Material and methods

To perform the cytogenetic study, 10 larvae of *Melitoma segmentaria* within the nest cells were collected in Viçosa – Minas Gerais, Brazil (20°44'58.03"S, 42°51'8.98"W). We sampled 10 individual nests. The cells were opened in the laboratory to verify the larval stage. The larvae that were not at the post-defecation stage were maintained in a biological oxygen demand (BOD) chamber (Marconi, model MA-415/S), at 25°C, until they reached the appropriate stage.

The metaphase chromosomes were obtained from the larval cerebral ganglia in the post-defecation stage (Imai et al. 1988). If the ganglia were large enough, they were divided into two or more sections. Chromosome characterization was performed by conventional Giemsa staining and C-banding (BSG method: Barium hydroxide (5%)/saline solution (2XSSC, pH 7.0)/Giemsa (8%)), as reported by Sumner (1972). Sequential staining with fluorochromes chromomycin A_3_ (CMA_3_) and 4’,6-diamidino-2-phenylindole (DAPI) was carried out according to the methodology of Schweizer (1980). The technique Ag-NOR presented by Howell and Black (1980), was used for the location of the NOR.

The metaphases were analyzed with the aid of an Olympus BX 60 microscope coupled to an image capturing system, Q Color3 Olympus^®^. For analysis of the fluorochromes, WB filters (450 – 480 nm) were used for CMA_3_ and WU filters (330 – 385 nm) for DAPI. The karyotypes were assembled according to the classification established by Imai (1991), which took into consideration the heterochromatin pattern.

## Results and discussion

**Figure 1.**
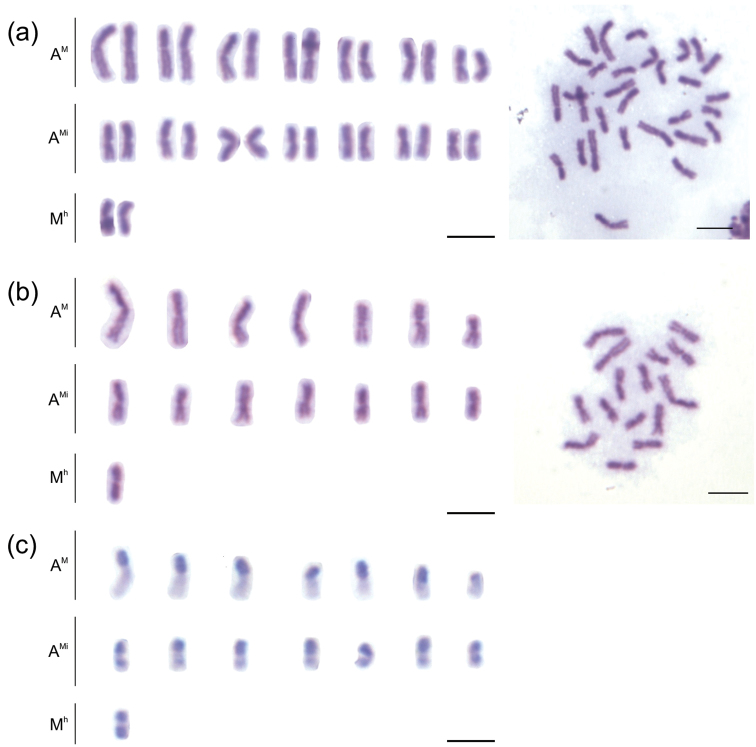
Mitotic karyotypes of *Melitoma segmentaria*. **a** Giemsa staining (female) **b–c** C-banding (male). Bar=5μm.

The species *Melitoma segmentaria* showed a chromosome number of 2n=30 for females and n=15 for males (Fig. 1). This chromosome number was similar to that observed in other solitary bee species, including *Ceratina megastigmata* Yasumatsu and Hirashima, 1969 (2n=34), *Xylocopa appendiculata* Smith, 1952 (2n=32), and *Pithitis smaragdula* (Fabricius, 1787) (2n=28) (Hoshiba and Imai 1993). However, it was lower than the value found in *Euglossa*, that is, 2n=42 (Fernandes et al. 2013).

The C-banding technique allows the observation of large positive heterochromatic blocks in the chromosomes of *Melitoma segmentaria* (Fig. 1c), wherein, at least one of the arms, has been completely heterochromatic. Taking into account the C-banding pattern and the nomenclature proposed by Imai (1991), the chromosomes can be classified into three different types: seven pseudo-acrocentric pairs (A^M^) with one heterochromatic arm, seven pseudo-acrocentric pairs with an interstitial heterochromatin (A^Mi^), and one totally heterochromatic metacentric pair (M^h^) (see Fig. 1c). According to Imai (1991), pseudo-acrocentric chromosomes are the result of a centric fission, followed by a significant addition of heterochromatin in the telomere region, in order to restore the stability of the chromosome. The entirely heterochromatic metacentric pair may arise from the centric fusion of two heterochromatic acrocentric chromosomes (A^h^). A fully heterochromatic metacentric chromosome is uncommon, and this morphological type is found in some supernumerary and Y-chromosomes (Imai 1991, Costa el al. 1992, Camacho et al. 2000, Lopes et al. 2008). All individuals analyzed, both females and males, possess this entirely heterochromatic chromosome, which indicates that it is a part of the autosome complement, and hence, it has not been treated as a supernumerary chromosome.

The pattern of heterochromatin distribution in *Melitoma segmentaria* is similar to that observed in most of the studied Meliponini species (Rocha et al. 2003, Carvalho and Costa 2011, Miranda et al. 2013), where most of the chromosomes in the complement have a single heterochromatic arm. This seems to agree with the “minimum interaction hypothesis,” proposed by Imai et al. (1988), as the main mechanism of karyotype evolution in these bees. According to this hypothesis, one metacentric chromosome breaks apart at the centromere producing two acrocentric chromosomes. Therefore, due to the instability of these acrocentric chromosomes, the repetitive DNA starts an *in-tandem* growth at the telomere region, leading to chromosomes with a heterochromatic arm (see Imai et al. 1988), as observed here in *Melitoma segmentaria*. However, this pattern is very different from that observed in the solitary bee *Euglossa carolina* (Linnaeus, 1758) (Fernandes et al. 2013), suggesting that alternative mechanisms of karyotype change may occur through the evolutionary diversification of these species. More detailed karyotype studies are needed to point out the trend in the karyotype evolution of solitary bees.

**Figure 2.**
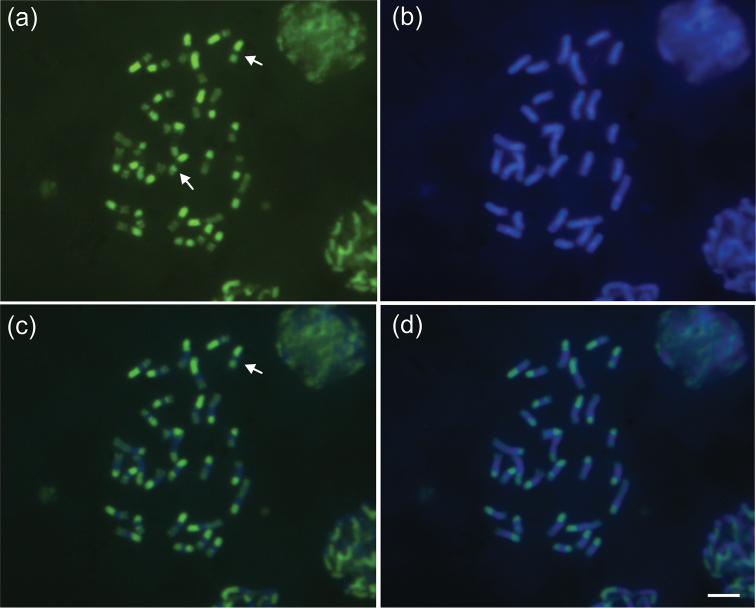
Female mitotic karyotypes of *Melitoma segmentaria* stained with fluorochromes: **a** CMA_3_ **b** DAPI **c** CMA_3_/DAPI and **d** DAPI/CMA_3_. Arrows indicate entirely heterochromatic metacentric chromosomes (M^h^). Bar=5μm.

Chromosome staining with the fluorochromes CMA_3_ and DAPI (Fig. 2) shows that heterochromatin has an apparently homogeneous constitution. However, the fluorochrome CMA_3_ shows that the heterochromatin present in the chromosomal arms of *Melitoma segmentaria* is more GC-rich than AT-rich. DAPI in *Melitoma segmentaria* marked the centromeric and pericentromeric regions of the chromosomes, indicating that these regions are rich in AT base pairs. In Meliponini bees the heterochromatin is rich in AT base pairs (it is therefore DAPI^+^) (Brito et al. 2003, Rocha et al. 2003, Lopes et al. 2008). The karyotype of *Melitoma segmentaria* shows heterochromatin richness that is different from the eusocial bees. A similar result has also been observed by Fernandes et al. (2013) in the bee *Euglossa carolina*. Taken together, these results suggest that the evolution of repetitive DNA, the main component of heterochromatin, evolves in different ways in social and solitary bees. However, this conclusion must be treated with caution, because data on only two solitary bees are available and this needs further evaluation.

**Figure 3.**
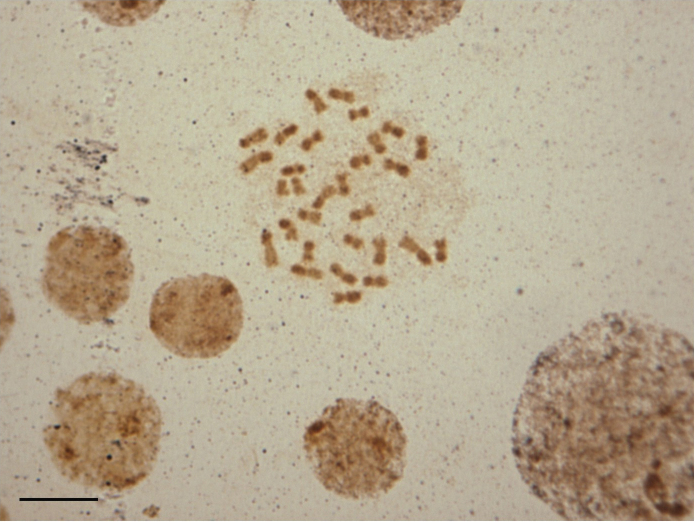
Female mitotic chromosomes of *Melitoma segmentaria* submitted to silver-nitrate staining. Dark regions on the heterochromatin arms indicate silver staining. Bar=5μm.

In order to identify the position of the NORs in the genome of *Melitoma segmentaria*, impregnation with silver nitrate was performed (Fig. 3). However, the methodology used was not efficient enough to indicate the location of the NOR. A particular pattern found in the chromosomes of *Melitoma segmentaria* was a result of silver staining of the heterochromatic chromosomal arms. Overall, NORs were associated with the GC-rich regions, as observed in the bee genus *Friesella* Moure, 1946 (Mampumbu and Pompolo 2000), *Partamona* Schwarz, 1939 (Brito-Ribon et al. 2005, Martins et al. 2013), and *Melipona* Illiger, 1806 (Maffei et al. 2001). The relationship between the NORs and the CG-rich regions was also suggested for the other Hymenoptera species (Cardoso et al. 2012). The recurrent relationship between CMA_3_^+^ and Ag-NOR staining was also observed in the present study, but the positive Ag-NOR staining in the heterochromatic regions of the diploid chromosome set, was unlikely to indicate the actual position of the NOR. Multiple positive Ag-NOR staining, coincident with C-banding and CMA_3_^+^ staining, was reported for the stingless bee *Scaptotrigona xanthotricha* (Duarte et al. 2009), and now here for *M. segmentaria.*

The silver impregnation technique located the NOR by staining the proteins present in this region. Sumner (1990) reported that this method was used to visualize heterochromatic regions that were not associated with NORs in various organisms. Therefore, this suggested that the proteins associated with the NORs were qualitatively similar to those encountered in the heterochromatic blocks of *Melitoma segmentaria*. Our results raised issues about the entire effectiveness of Ag-NOR staining, to correctly identify the NOR in all taxa. Future studies, using specific probes for NORs, by means of the fluorescence *in situ* hybridization (FISH) technique, might help to elucidate this.

This study is the first detailed karyotype characterization of the *Melitoma* species, bringing to light several chromosome features, such as, chromosome number, morphology, heterochromatin pattern, and base pair richness. Characterizations of the karyotype of other species of solitary bees and of the genus *Melitoma*, coupled with the use of banding and staining techniques are needed, to obtain a better understanding of the chromosomal evolution in Apidae.
